# Pyrene-based hole transport materials for efficient perovskite solar cells

**DOI:** 10.55730/1300-0527.3727

**Published:** 2025-01-04

**Authors:** Madiha IRFAN, Aamer SAEED, Sania TAHIR, Muhammad Rehman YAQOOB, Sohaib Ahmed ZIA, Muhammad USAMA, Junaid MUKHTAR, Usama FARRUKH, Sara REHMAN

**Affiliations:** 1 Institute of Chemistry, Khawaja Fareed University of Engineering and Information Technology, Rahim Yar Khan, Pakistan; 2 Department of Chemistry, Faculty of Natural Sciences, Quaid-i-Azam University, Islamabad, Pakistan

**Keywords:** Pyrene, pyrene-based HTMs, perovskite solar cells, power conversion efficiency, hole transport materials

## Abstract

In the past few years, perovskite solar cells (PSCs) have gained a lot of attention and become a well-known topic in solar studies due to their lower manufacturing costs and improved efficiencies. Previously utilized hole transport materials (HTMs), such as poly[bis(4-phenyl)(2,4,6-trimethylphenyl)amine] and 2,2′,7,7′-tetrakis(N,N-di-p-methoxyphenylamine)-9,9′-spirobifluorene, are challenging due to their high price, complicated synthesis, limited carrier mobility, and poor device stability. Developing HTMs for PSCs that are inexpensive and high-performance has gained much interest. Currently, many HTMs of organic molecules are used to improve photovoltaic qualities and reduce synthesis costs. Effectively using HTMs is essential for producing the best photovoltaic efficiency in PSCs because they are essential in extracting and transporting charge carriers. Pyrene-based HTMs have excellent device performance, chemical stability, and photovoltaic qualities compared to the other organic moieties. The significant developments made in pyrene-based HTMs over the past five years are reported herein. This review analyzed the relationship between the molecular structure, hole mobility, highest occupied molecular orbital–lowest unoccupied molecular orbital energy levels, power conversion efficiency (PCE), and energy band gap of pyrene-based HTMs. It was revealed that PSC devices fabricated with pyrene-based HTMs have attained a PCE greater than 22%. It is hoped that this review will encourage more researchers to develop HTMs that have good performance, low cost, and high device stability.

## 1. Introduction

Renewable energy, particularly solar energy, has become a sustainable and environmentally favorable replacement for fossil fuels [1]. The most abundant carbon-free energy source is solar energy, which can be converted into heat and electricity. Photovoltaic cells are the most efficient for direct conversion of sunlight into electrical power. Initially, silicon-based and inorganic solar cells with high levels of efficiency were developed. More recently, hybrid perovskite solar cells (PSCs) made of organic-inorganic halides fascinated optoelectronic fields because of their unique photovoltaic attributes [2]. PSCs have power conversion efficiencies comparable to crystalline silicon solar cells, establishing them as an emerging photovoltaic technology [3]. PSCs have shown incredible advancements from a starting efficiency of 3.8% in 2009, with their power conversion efficiency (PCE) now exceeding 25% [4].

In PSCs, there are three distinct cation types: monovalent ions, denoted by A; the bivalent charge of a metal component, denoted by M; and the halide ion, denoted by X. A PSC device design usually has five parts: a transparent conductive substrate made of fluorine-doped tin oxide or indium tin oxide, a barrier that transports electrons, a hole-carrying membrane, perovskite, and metal electrical contacts made of gold or silver [5]. Each interface incorporates layers for transporting electrons and holes to facilitate the transfer of ions and collections [6]. The arrangement of charge transport layers determines the device type classification, resulting in two categories: inverted and conventional structures [7].

**Figure 1:**
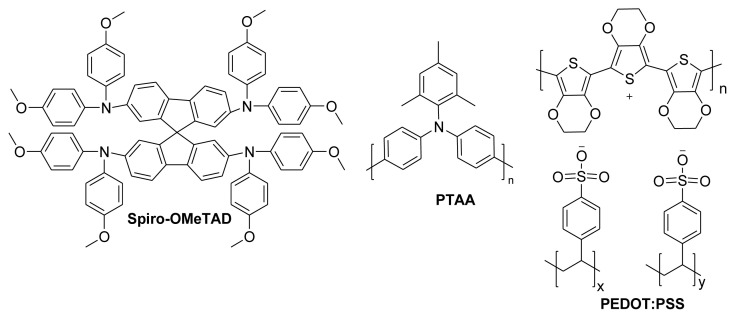
Small molecule and poly molecule used as the HTMs.

Hole transport materials (HTMs) are an important part of PSCs because they lower charge recombination and make it easier for the cells to convert light into electricity [8]. The converting power of a photovoltaic tool is affected by both the hole/electron carrying materials and the light-harvesting ability of the sensitizers. Photovoltaic systems usually comprise light-absorbing components positioned between the layers that handle the movement of electrons and holes [9]. Organic small molecules and polymers, and inorganic salts are commonly used HTMs [10]. Small-molecule HTMs, selected the as standard, are called 2,2′,7,7′-tetrakis(N,N-di-p-methoxyphenylamine)-9,9′-spirobifluorene or Spiro-OMeTAD (Figure 1). Initially introduced by Li et al. [11], this compound has been established as the benchmark HTM due to its consistent and outstanding performance. The charge extraction procedure relies heavily on the HTM layer and influences the stability of the PSC [12]. Spiro-OMeTAD is not a feasible alternative because of its instability and high price [13]. Alternatives cheaper than spiro-OMeTAD must be found to commercialize hybrid PSCs. The most prominent polymeric HTMs are poly[bis(4-phenyl)(2,4,6-trimethylphenyl)amine] (PTAA) and poly(3,4-ethylenedioxythiophene)-poly(styrenesulfonate) (PEDOT:PSS) (Figure 1). Despite its greater performance, PTAA is costly and requires the addition of dopants. One major disadvantage of PEDOT:PSS is its acidity, which reduces its chemical stability [14].

The main requirements of HTMs, including excellent stability, simple synthesis, affordability, and increased hole mobility (μ_h_), have yet to be satisfactorily addressed [15], making their fulfillment a significant challenge in PSC research.

**Figure 2:**
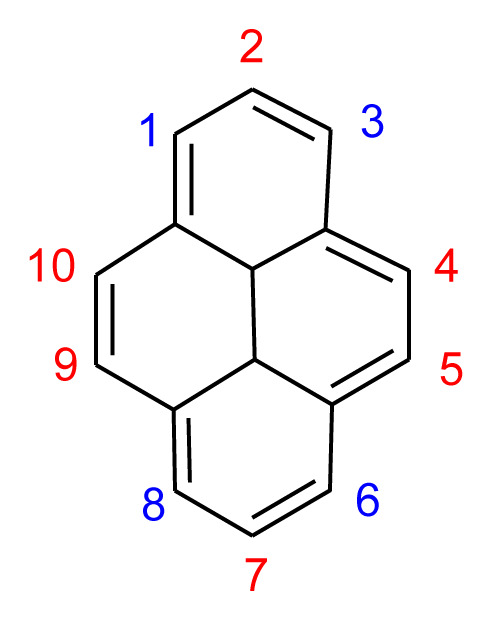
Molecular structures of the pyrene with respective positional numbering.

Hence, pyrene-based hole-transport layer (HTL) materials deserve attention. Pyrene is composed of four joined rings of benzene in a replacing aromatic polycyclic hydrocarbon [16]. Planar-fused pyrene-based conjugated materials (small molecules and polymers) exhibit excellent charge transport and photochemical properties. The availability of reactive sites on the pyrene core allows for easy substitution with appropriate functional groups, making it a fascinating benefit [17]. The common sites for derivatization are often positions 1, 3, 6, and 8 of pyrene, which can be easily altered to create mono-, di-, tri-, and tetra-substituted pyrene derivatives because of their high reactivity. However, the reactivity of the other uncommon sites of pyrene, positions 2, 4, 5, 7, 9, and 10, is comparatively lower (Figure 2) [18]. Pyrene can move charges quickly because its electrons are not all in the same place and it has excellent thermal stability, with some glass transition points above 300 °C [19]. Numerous pyrene derivatives have been created to date and employed in a range of devices, including solar cells, organic light-emitting diodes, organic field-effect transistors, and light-emitting electrochemical materials [20]. Recent literature reported the effects of the substituents of pyrene-based HTL materials on PSC performance [21].

This review paper will summarize the recent developments in pyrene-based compounds in PSCs and discuss their growth and commercialization challenges. Furthermore, a comparative investigation will examine the efficiency and stability of several pyrene-based compounds used in PSCs by different researchers.

## 2. Pyrene-based HTMs

**Figure 3:**
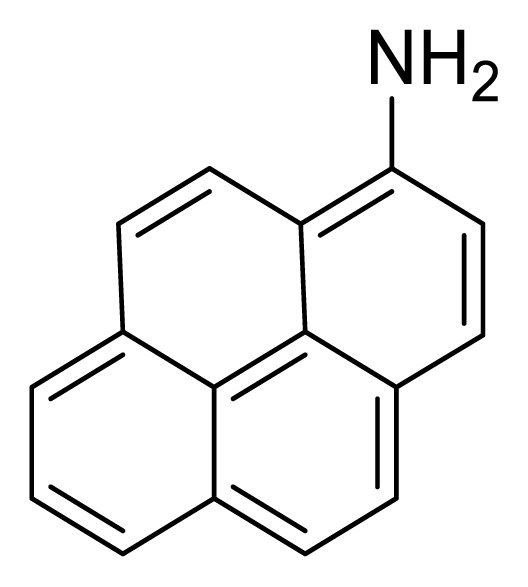
Structure of the AMP.

De et al. [9] investigated the capability of 1-ampinopyrene (AMP) (Figure 3) to extract holes from commonly used nanocrystals (NCs) that are all-inorganic perovskites (CsPbI_3_ and CsPbBr_3_), with differing band gaps and characterized them using transmission electron microscopy. CsPbI_3_ had a typical lifetime of about 4.18 and 5.9 ns for CsPbBr_3_, corresponding to the earlier literature [22]. The quantum yield photoluminescence (PL) of about 68% for CsPbBr_3_ and 60% for CsPbI_3_ NCs were recorded, and a dramatic drop in the PL of these NCs was observed with the subsequent addition of AMP.

The energy of conduction and the valance band edge were estimated in a previously conducted study [23]. The energy of the highest occupied molecular orbital (E_HOMO_) and energy of the lowest unoccupied molecular orbital (E_LUMO_) for AMP were measured as −5.04 and −1.95 eV, respectively, determined by cyclic voltammetry (CV). The hole transfer periods for these NCs were calculated as 120 and 170 ps, respectively, equal to or better than those measured by Wu et al. [24] (26 and 330 ps) and Sarkar et al. [25] (120–170 ps). Taking advantage of the pi bond framework of pyrene and the exceptional surface anchoring capabilities of perovskite NCs, it was demonstrated that even a fundamental system like AMP could be used with favorable hole transport properties.

From these findings, researchers believed that the photoelectric conversion efficiency (PCE) of perovskite NCs could be enhanced by fabricating these solar cells with a pyrene core, to which appropriate functional groups would be attached, and they could then be utilized as cheap and highly efficient HTMs.

Ge et al. [26] reported a small organic molecule called OMe-TATPyr [1,3,6,8-tetrakis[5-(N,N-di(p-methoxyphenyl)amino-p-phenyl)-thiophen-2-yl] (1), as a very effective HTM for PSCs. The OMe-TATPyr molecule, with phenyl-thiophene components, enhanced the mobility of carriers and the capacity of the molecules to stack on top of one another to improve the delocalization of charges. It also allowed the creation of fewer adhesive traps through the interaction between lead and sulfur, which would make it an efficient and better choice for futuristic PSCs. The two-dimensional pi-conjugated structure of the OMe-TATPyr was characterized by phenyl-thiophene bridged triarylamine units attached to the pyrene-base core. Density functional theory calculations were performed to measure the torsional angles between the pyrene-base core and the phenyl-thiophene groups, which were anticipated to be greater than 40°.

In contrast to the E_HOMO_ (−5.2 eV) value for spiro-OMeTAD, [27] OMe-TATPyr (1) was reported to have an E_HOMO_ of about −5.4 eV and E_LUMO_ of −3.1 eV, which is considerably higher than the conduction band (−3.8 eV) of perovskite. Thermogravimetric analysis (TGA) was used to determine that Py-C disintegrated at about 400 °C, whereas OMe-TATPyr started to break down at roughly 450 °C. These results revealed the exceptional heat stability of these pyrene derivatives.

The mean short circuit current density (J_sc_) of the OMe-TATPyr molecule was reported as 23.42 mA cm^−2^ and the μ_h_ value of the molecule (2.28 × 10^−4^ cm^2^ V^−1^ s^−1^) was more than that of spiro-OMeTAD (1.11 × 10^−4^ cm^2^ V^−1^ s^−1^) investigated under identical circumstances. Stability tests were also performed and it was noted that the device containing the OMe-TATPyr showed stability equivalent to the device with the spiro-OMeTAD. The highest recorded PCE for the OMe-TATPyr at a maximum of 20.6% was higher than that of the spiro-OMeTAD, which had previously performed in power conversion at 18.4%.

Chen et al. [21] reported two new small organic molecules (PTTI-1 and PTTI-2) (**11**) that were created by introducing the pyrene unit as the main building block and thieno[3,4-b]thiophene (TT) as the conjugated linking unit. The difference between the two molecules is the location of the sulfur atom in TT. The sulfur atom proved useful for improving PSC performance because it could create an interfacial S-I or S-Pb interaction to passivate the crystal surfaces of the perovskite. It increased the electron mobility, robust passivation behavior, and appropriate energy level, and PTTI-1 exhibited a higher power conversation efficiency (PCE) value of 15.37% than PTTI-2 (11.07%). According to their research, the performance of PSCs is significantly influenced by the sulfur location in this kind of electron-transport material. With the lambda max (λ_max_) at 638 and 612 nm, respectively, PTTI-1 and PTTI-2 both showed an apparent wide absorption between 500 and 700 nm. The absorption wavelength onset (λonset), on the other hand, was blue-shifted from 694 (PTTI-1) to 668 (PTTI-2) nm. The band gaps of PTTI-1 and PTTI-2 were 1.79 and 1.86 eV, respectively, using the equation Eg opt = 1240 nm / λonset. Additionally, PTTI-1-based devices exhibit good stability.

Through comprehensive research, Andijani et al. [28] reported eight derivatives of the parent compound (PYOMe) and divided them into two groups based on the nature of their substituents. Group A contained four compounds: PYA3, PYA2, PYA1, and PYOEt, and the remaining four compounds: PYOPh, PYOH, PYEt, and PYMe were assigned to the group B. The diaryl ring attached to the pyrene core had an N-atom in a different place in these compounds than the parent N1, N1, N3, N3, N6, N6, N8, N8-octakis(4-methoxyphenyl)pyrene-1,3,6,8-tetraamine (PYOMe). In contrast to the parent molecule with the −OMe group, R groups with different donation abilities of electrons were added (**2**), which revealed the molecular structures of the original PYOMe molecule and its derived ones. When compared to group B and the parent compound, the designed derivative in A (with strong electron donating groups) had a small Δ_H-L,_ longer λmax_abs_, higher f max_abs_, small λ_hole_, and lower adiabatic ionization potential (IP) values. As a result, it is regarded among the most promising HTMs to improve PSC performance.

The geometric and electrical properties of the studied HTMs in their ionic and neutral forms were assessed. The outcome of the frontier molecular orbitals (FMO) research revealed that PYOMe and its derived compounds had excellent hole-transport abilities. It was determined that the primary difference between the cationic, anionic, and neutral forms was in the relative magnitude of the bond angle and dihedral angle. However, these values increased and decreased in these forms, respectively. For example, the change in the bond angle between the cationic and neutral forms for the proposed compounds ranged from 3.2° to 5.2°, and the PYOPh bond angle value was estimated to be 5.5°. Likewise, the values for the dihedral angle varied from 16.3° to 22.2° for the remaining molecules, and for PYOPh, it was determined to be 23.6°. The FMO analysis indicated that the HOMO was virtually spread over PYOMe, exhibiting the π-features of the molecule. When acceptable E_LUMO–_E_HOMO_ levels were considered, molecules with ethoxy and amine R groups (derivatives of group A) appeared as the better options among many HTMs to increase the efficiency of PSCs when compared to PYOMe with derivatives in group B.

HTMs for PSCs that are derivatives of carbazole with 4,4’-dimethoxydiphenylamine (DMPA) units have recently been presented [29,30]. In a single-step process, two-HTMs based on carbazole with two or four fused benzene rings show the structures of the newly synthesized molecules that are made with units of pyrene (Cz-Pyr) and naphthalene (Cz-N) attached to a 3,6-CzDMPA (3). The newly made devices constructed with Cz-Pyr had a PCE as high as 17.2% with no further optimization. Researchers believed that Cz-Pyr is an outstanding and cost-effective HTM [31] and synthesized the studied compounds in a single step from the commonly available commercial 3,6 CzDMPA precursor using the procedure of Buchwald–Hartwig cross-coupling reaction utilizing a palladium catalyst. A TGA study showed that these two HTMs demonstrated high thermal stability. The degradation temperature was considered appropriate for the PSC used (432 °C for Cz-Pyr and for Cz-N, 425 °C). The glass transition temperature (Tg) for Cz-N and Cz-Pyr was measured to be 107 and 109 °C, comparable to spiro-OMeTAD. Each HTM’s IP was determined using CV. All the examined HTMs had comparable IP values estimated by CV, (−5.19 eV for Cz-N, −5.18 eV for Cz-Pyr, and Spiro-OMeTAD with an IP value of −5.20 eV vs. the vacuum).

The spiro-OMeTAD had optical band gaps of −2.99 eV, while the Cz-Pyr as well as Cz-N band gap values were recorded as −2.86 and −3.05 eV, which were calculated from the absorption onset. With good μ_h_ (7.4 × 10^−6^ cm^2^ V^−1^ s^−1^ for Cz-Pyr and 1.0 × 10^−5^ cm^2^ V^−1^ s^−1^ for Cz-N), were analogous to Spiro-OMeTAD observed under the same circumstances (2.5 × 10^−5^ cm^2^ V^−1^ s^−1^) [32]. Cz-Pyr-based PSCs frequently deliver much stronger photovoltaic performance than spiro-OMeTAD-based devices, primarily owing to a higher J_sc_. In contrast to spiro-OMeTADs 13.7%, the devices attained 17.2% PCE using Cz-Pyr as the HTM without further tuning. The potential use of extendable fused benzene rings as substituents (i.e. pyrene) on HTMs with a carbazole-base was demonstrated by the present work.

The compound 1,3,6,8-tetrakis[N-(p-methoxyphenyl)-N’-(9,9’-dimethyl-9H-fluoren-2-yl)-amino]pyrene (TFAP) (4) was investigated by Shao et al. [33], and the PCE was 19.7%. They showed that the presence of the fluorene group significantly enhanced the TFAP ability to extract and transport holes. The less electron-donating nature of the fluorene unit allowed the HOMO level of the TFAP to be stabilized, resulting in enhanced hole extraction. The more extensive conjugation structure of the fluorene unit may also have played a role in the movement of carriers from one molecule to another. The TFAP-based PSCs were just as efficient as the spiro-OMeTAD-based control device.

The reaction between N-(p-methoxyphenyl)-N’-(9, 9’-dimethylfluoren-2-yl) amine and 1,3,6,8-tetrabromopyrene led to the creation of TFAP. Based on the experimental findings, it was discovered that the E_HOMO_ of the spiro-OMeTAD was calculated as −5.22 eV relative to the vacuum. Similarly, the E_HOMO_ of the TFAP was −5.27 eV. The TFAP material would benefit the transfer of holes from the perovskite layer, as this study showed that using MAPbI_3_, a perovskite material with valence band energy calculated to be 5.43 eV.

The estimated value for the E_LUMO_ of the TFAP was −2.91 eV. The E_gap_ was calculated as 2.36 eV. The TFAP compound had an open-circuit voltage (V_oc_) of 1.11V, PCE_best_ of 19.7%, μ_h_ of 4.5 × 10^−3^ cm^2^ V^−1^ s^−1^, J_sc_ of 22.63 mA cm^−2^, FF of 74.05%, and decomposition temperature (T_d_) of 458 °C. The devices that used TFAP and spiro-OMeTAD had a PCE_avg_ of 18.60% and 18.67%, respectively.

Gomez et al.[34] studied two new compounds, pyrenodiindole (PDI) and pyrenodi-(7-azaindole) (PDAI) (6), which were introduced in Pb-Sn PSCs as HTMs. These compounds demonstrated improved durability, leading to a 16.1% increase in the PCE. The optical interference was changed in PSCs by using PDI and PDAI as HTL. According to research, it decreases unwanted absorption in the near infrared region, thereby increasing short-circuit current density. In addition, PDAI has a smaller difference between the E_HOMO_ and E_LUMO_, which indicates a good energy level alignment, leading to enhanced efficiency.

The synthesis of PDAI and PDI involved the Buchwald–Hartwig cross-coupling reaction, which was catalyzed by double palladium. This reaction was carried out between 1,6-diaminopyrene and the appropriate dihaloderivative, resulting in the synthesis of N-arylated intermediate. These intermediates engage in a photo-induced, regiospecific dual intramolecular cyclization process, producing the required polyheteroaromatic systems PDI and PDAI. From CV studies, the reported E_LUMO_ levels for PDI and PDAI were −2.82 and −3.22 eV, and for E_HOMO_ they were −5.35 and −5.54 eV, respectively. PDAI and PDI demonstrated the same band gap of approximately 2.32 eV. The PCE for PDI was 16.1%, while the charge transit time was 0.96 μs, J_sc_ was 29.1 mA cm^−2^, T_d_ was 443 °C, V_oc_ was 0.78 V, access to the hole value was 2.7 × 10^−3^ cm^2^ V^−1^ s^−1^, and fill factor (FF) was 0.71.

The PDAI displayed a hole movement of 2.9 × 10^−3^ cm^2^ V^−1^ s^−1^, V_oc_ of 0.77 V, charge transit time of 0.90 μs, and density component (FF) of 0.71. The T_d_ was measured at 534 °C and the J_sc_ at 29.1 mA cm^−2^. The total PCE was 16.1%.

Shao et al.[35] revealed conjugation to be the driving force behind the higher efficiency of a star-shaped monomer called poly-17 (**9**) with four triphenyl side arms, which was synthesized by electropolymerizing 1,3,6,8-tetra(5-bromothiophen-2-yl)pyrene. An impressive 16.5% PCE was attained in inverted PSCs by employing poly-17 films as the HTM. The strong hydrophobicity and linked structures of the poly-1 films were responsible for its efficiency.

The synthesis of the poly-1 monomer was achieved using a Suzuki coupling reaction involving 1,3,6,8-tetra(5-bromothiophen-2yl)pyrene and 4(diphenylamino)phenylboronic acid. It exhibited an E_HOMO_ of −5.4 eV. To determine how the depth of the film affected how the gadget worked, several poly-1 films were made by altering the patterns of potential scan cycles throughout the electropolymerization process. Poly-17 demonstrated the most favorable outcomes, having been synthesized through 10 possible scan cycles. The film exhibited a PCE of 16.5%, V_oc_ of 0.99 eV, FF of 0.69, short circuit current density of 22.7 mA cm^−2^, and movement of the hole of 1.0 × 10^−5^ cm^2^ V^−1^ s^−1^.

Tepliakova et al. [36] investigated the structures and characteristics of four pyrene derivatives and concluded that pyrene derivative Y2 (5) with a naphthyl substituent produced the most optimistic results, with a PCE of 17.9%. The substitution pattern in the Y2 compound provided perfect molecular geometry, resulting in dense clustering in the solid phase and enhanced charge transfer attributes. The enhanced efficiency of Y2 was due to its perfect molecular geometry and high charge transport characteristics.

The electrochemical characteristics of the Y2 were studied using CV. The E_HOMO_ level was −5.5 eV and the E_LUMO_ was −2.8 eV. The E_gap_ was 2.6 eV. Y2 exhibited a μ_h_ of 5 × 10^−3^ cm^2^ V^−1^ s^−1^, melting point of 290 °C, FF of 78%, Td of 460 °C, and J_sc_ of 21.2 mA cm^−2^.

The innovative study conducted by Ou et al. [37] emphasized the crucial role of conjugation in achieving unrivaled efficiency in the SY3 compound (**2**). CsPbI2Br and (FAPbI3)_0.85_ (MAPbBr3)_0.15_-centered PSCs achieved a PCE of 13.41% and 19.08%, respectively, with SY3-based HTMs. HTM-SY3 with a pyrene-bridge demonstrated increased μ_h_, conjugation, central π-bridge composition, thermal stability, enhanced hole extraction/transport, improved film formation capabilities, and durability.

They substituted phenanthrene with a pyrene unit, serving as the central π-bridge, resulting in SY3-centered PSCs that demonstrated noteworthy attributes. The E_LUMO_ and E_HOMO_ were calculated as −2.74 and −5.25 eV. The SY3-based (FAPbI_3_)_0.85_ (MAPbBr_3_)_0.15_ exhibited a FF of 75.7%. V_oc_ of 1.114 V, and J_sc_ of 22.62 mA cm^−2^, maintaining PCE at 19.08%.

The SY3-based CsPbI_2_Br PSCs produced a PCE of 13.41% with a movement of hole of 2.18 × 10^−4^ cm^2^ V^−1^ s^−1^, E_gap_ of 2.51 eV, V_oc_ of 1.19V, hole reorganization energy (E_R_) of150 meV, FF of 75.5%, and J_sc_ of 14.93 mA cm^−2^. The T_d_ of SY3 was determined as 382 °C. When operating at the maximum power point, the SY3-based PSCs showed high performance and maintained a PCE of 18.95%.

By introducing a fluoro-substituted pyrene into polymer chains of thiophene and selenophene, Yao et al. [38] studied a new family of polymeric HTMs. PE10 (**8**) showed the highest efficiency of all the discussed compounds, with an excellent PCE of 22.3%. To improve the charge transport, HTM molecule stacking was bolstered by expansive entangled planes composed of pyrene and Se atoms, which also significantly linked with the perovskite surface. Additionally, fluorine replacement close to the pyrene shifted the HTM stacked to a more beneficial face-on orientation, which improved the charge transmission. In addition, PE10 had a shorter-stacking distance, which helped to contribute to the gradually expanded hole mobilities, which in turn led to better charge transfer and optimal FFs in the PSCs that were produced as a result.

This work described a synthesis of a currently discovered HTMs by introducing a fluoro-substituted pyrene into chains of polymers of thiophene and selenophene. Dopant-free HTMs were created by adding pyrene to chains of polymers of thiophene and selenophene. By replacing some of the pyrene with fluorine atoms, the HTM’s energy ranges and geometric shapes may be adjusted [38].

PE10 also exhibited a shorter-stacked distance of 4.3Å compared to PE7 before the fluorine substitution, which was 4.5Å. Increased charge transfer in the substance used to carry hole levels and better FF in successive PSCs were made possible by PE10’s access hole movement of 1.46 × 10^−3^ cm^2^ V^−1^ s^−1^ [39]. The polymer film’s HOMO, calculated from the CV, was −5.34 eV for PE10; this value was generally in agreement with that calculated from UV photoelectron spectra. Maximum energies of occupied molecular orbitals in four distinct polymers provided sufficient force to push the hole extractions, in contrast to the perovskite E_HOMO_ power level of −5.43 eV.

In comparison to the NiOx-only cell’s PCE of 18.66%, the NiOx/CL-3 dependent cell (**7**) had the highest PCE, at 20.15%. NiOx/CL-3 had higher efficiency as a result of its streamlined surface, better exchange of charges, and uniform alignment for energy levels. The CL-3 molecule was the most efficient because its E_LUMO_ level was the lowest. This was good for moving electrons. Moreover, this device was less water-repellent and had good long-term stability [40].

The required compound was synthesized with a pyrene-based diarylamine process. Because of its extra conjugation pyrene unit’s proximity to the center of the molecule, compound CL-3 exhibited an increased factor for absorption and more reddish shift than the others [41]. The CL-3 compound had a peak emission band at about 528 nm. The E_HOMO_ at level CL-3 was −5.39 eV, which was remarkably close to the perovskite level (−5.4 eV) [42]. The E_LUMO_ at level CL-3 was −2.78 eV. The LUMOs were also found to change as new diarylamines units were introduced into the system.

The E_gap_ was also determined to be within a range of 2.38–2.77 eV. The CL-3 had a T_d_ value, which was significantly greater than that of other materials because the pyrene ring was highly stiff and bulky [43]. The largest Tg was seen in the pyrene-based arylamine (CL-3), since bigger pyrene substituents impeded intramolecular rotations [44]. Root mean square values for spin-coated (nickel oxide) × and NiOx/CL-3 sheets were 3.16 and 1.99 nm, respectively.

PSCs using the undoped pyrene fluoride (PFD) (**10**) had a PCE of 18.6%. The PCE rose to 21.02% while spiro-OMeTAD was incorporated into PFD (PFD/spiro-OMeTAD), but the steady-state efficiency fell to 19.92% [45]. According to the study, PFD had an almost two-dimensional structure and higher movement of the hole. In addition, a fundamental unit of the PFD was a rigid and planar pyrene group, and its arrangement is symmetric to enhance the pairing degree and facilitate the transport of carriers. Because of these qualities, PFD could be used in PSCs used as HTM that do not need dopants. It may additionally serve as a connection layer to stop the recycling of carriers, which makes it more effective than spiro-OMeTAD, which has not been doped.

Pyrene is the main building block for PFD, and fluorene is the link between triphenylamine and pyrene. Due to an internal charge-transfer shift from the substance known to the pyrene center, PFD ingested some visible light. The peak absorption was strongest at 383 nm (e_383 nm_ = 1.018105 L mol^−1^ cm^−1^), and the peak in the middle was at 430 nm (e_430 nm_ = 3.76104 L mol^−1^ cm^−1^) [33]. In steady-state photoluminescence, the largest emission peak of PFD can be seen at 532.4 nm. Ultraviolet-visible spectroscopy and cyclic voltage measurement showed that PFD had a band gap of 2.390 eV, that the peak of closed molecule gaps (E_HOMO_) grade was −5.20 eV, and its smallest unoccupied molecular spacing (E_LUMO_) level was −2.81 eV [46].

The movement of holes in untreated PFD was determined to have a value of 3.65 × 10^−5^ cm^2^ V^−1^ s^−1^. This was less than the μ_h_ of 8.49 × 10^−3^ cm^2^ V^−1^ s^−1^ reported in doped spiro-OMeTAD [47]. The dopant-free PFD was thought to have a conductance of 8.19 × 10^−5^ S m^1^ in the conventional Ohmic regime [48]. PFD may replace the HTM portion of the PSC because it has the same energy level as both the perovskite (VB: −5.40 eV) and the PSC (E_HOMO_: −5.20 eV), current intensity for scavenging was 22.81 mA cm^−2^, electric potential 1085 mV, and capacity factor 0.751, cells within the PFD level at 7 mg mL^−1^ achieved a PCE of 18.60% [49].

Li et al. [50] investigated the SMe-TATPyr (**13**), a sulfur-rich compound that was used as a dual-functional substance to increase the longevity and efficiency of PSCs. Their work showed that PSCs containing the SMe-TATPyr have a PCE of 22.3%. When SMe-TATPyr was used at the perovskite interface, extracting and transferring holes was easier. This was because SMe-TATPyr has enough sulfur atoms, which successfully passivated interface defects.

SMe-TATPyr was synthesized with a 40% yield through a reaction between 5-(N,N-di(*p*-(methylthio)phenyl)amino-*p*-phenyl)thiophene-2-boronic acid and 1,3,6,8-tetrabromopyrene. The E_HOMO_ and E_LUMO_ of the SMe-TATPyr were −5.5 and −3.2 eV, respectively [26], and the E_gap_ was 2.3 eV, utilizing the initial wavelength of absorption.

In CH_2_Cl_2_, it showed a prominent peak for the absorption band (460 nm) and the emission band (569 nm) [49], recorded the T_d_ of the SMe-TATPyr required for a 5% reduction in weight at 430 °C, and after 1500 h of storage at ambient temperatures, maintained 95% of its original efficiency. It exhibited a V_oc_ of 1.10 to 1.13 V, μ_h_ of 1.0 × 10^−3^ cm^2^ V^−1^ s^−1^, and hysteresis index (HI) of 9.8% to 5.9%. When the SMe-TATPyr interlayer was used in the PSC, the PCE_best_ increased from 20.40% to 22.34%.

The highest PCE (19.34%) was observed in the compound HY6 (6,6′, ′’,6′”-(pyrene-1,3,6,8-tetrayl)tetrakis(*N*,*N*-bis(4-methoxyphenyl)naphthalen-2-amine) (12) [51]. Because of increased planarity, stronger conjugation, and high charge mobility. It enhanced the hole-transporting capacity by promoting the movement of π-electrons. Therefore, HY6 achieved the highest possible efficiency.

The Suzuki–Miyaura reaction was used to synthesize HY6 [51] and it was found that the E_HOMO_ of HY6 (−5.35 eV) was greater than the valence band energy (−5.60 eV) of perovskite [51]. Perovskite materials CB (−3.90 eV) were lower than their E_LUMO_ (−2.76 eV; HY6), which can stop electrons from moving from the perovskite material. Furthermore, HY6 had an E_gap_ of 2.59 eV, emission wavelength of 526 nm, absorption wavelength of 420 nm, and λ_onset_ of 479 nm. The HY6 molecule underwent a significant structural change from S_0_ to S_1_ state, with a Stokes shift of 103 nm [52].

The best PCE for HY6-based PSCs was 19.34%, with a μ_h_ of 1.62 × 10^−4^ cm^2^ V^−1^ s^−1^, V_oc_ of 1.078 V, T_d_ of 443 °C, J_sc_ of 23.00 mA cm^−2^, and density component of 78.05%. Smaller HI values (HY6: 3.77%) suggested that HY6-based devices have greater hole extraction and transport.

The N,N-di-p-methoxyphenylamine substituted pyrene derivatives, specifically referred to as Py-A, Py-B, and Py-C, were also investigated. Jeo et al.[16] reported that the PCE of the third derivative among the three was 12.4%. The Py-C derivatives had performance comparable to the extensively researched spiro-OMeTAD. Moreover, they believed that the newly made pyrene compound (Py-C) had viability as a carrier of holes in inefficient solar cells. Using the CV results and the ultraviolet/visible spectrum, the E_HOMO_ and E_LUMO_ of the Py-C were determined as −5.11 and −2.74 eV, respectively. Therefore, the E_gap_ was 2.36 eV, and they reported that Py-C, a pyrene derivative, had a maximum absorption wavelength of 492 nm, short-circuit current density of 20.2 mA cm^−2^, density component of 69.4%, and V_oc_ of 0.886 V, series resistance of 51.57, and resulted in 12.4% overall efficiency.

This work reported the synthesis of four new chemicals for usage as HTL in PSCs, two of these centered on BODIPY-pyrene-triarylamine (BODIPY-boron-dipyrromethene) [53]. The power conversion rate of 18.80% was achieved when TM-04 was included in PSCs. Enhanced electronic communication and PCE were due to its strong conjugation and planar alignment.

TM-04 had a melting point of 327 °C. It had a 1.45 eV band separation with E_HOMO_ and E_LUMO_ of −4.83 and −3.38 eV, respectively. It was calculated that the movement of holes was 16.9 × 10^−5^ cm^2^ V^−1^ s^−1^. Reverse scanning of TM-04 revealed its V_oc_ to be 1.05V, its J_sc_ to be 22.27 mA cm^2^, and its FF to be 0.82, for a combined PCE of 19.12%. TM-04 had a V_oc_ of 1.04 V, current intensity for scavenging of 21.96 mA cm^2^, FF of 0.78, and PCE of 17.86% during the forward scan.

Pyrene-based derivatives with methoxy-substituted triphenylamine units (PyTPA, PyTPA-OH, and PyTPA-2OH) (**16**) were developed and synthesized by Wang et al. [54]. These derivatives had varying amounts of hydroxyl groups attached to the 2- or 2,7-positions of the pyrene core. By boosting the intermolecular interactions that can produce in situ radicals with the help of visible light irradiation, these hydroxyl groups at the 2- or 2,7-positions of pyrene significantly improved the hole-transferring ability, conductivity, and suppression of recombination.

**Figure 4:**
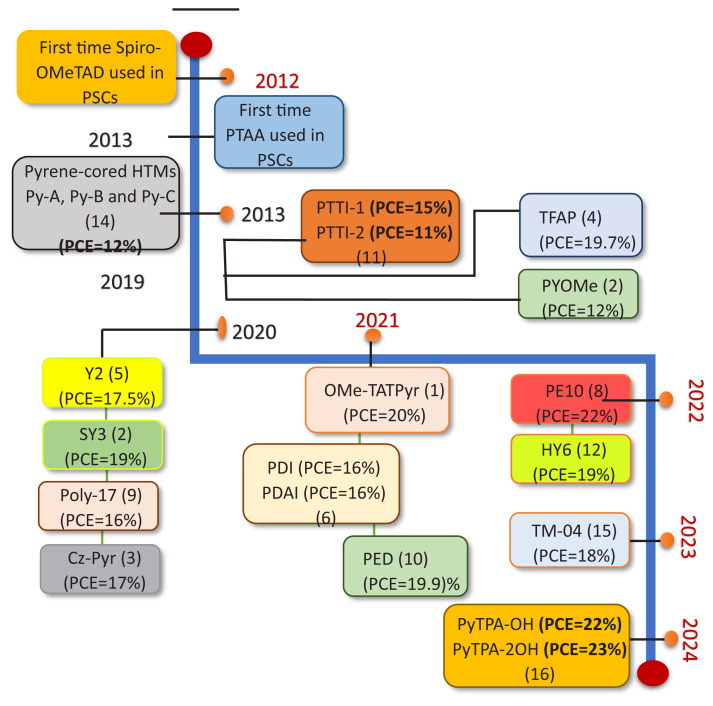
Efficiency evolution of the reported pyrene-based PSCs.

**Figure 5:**
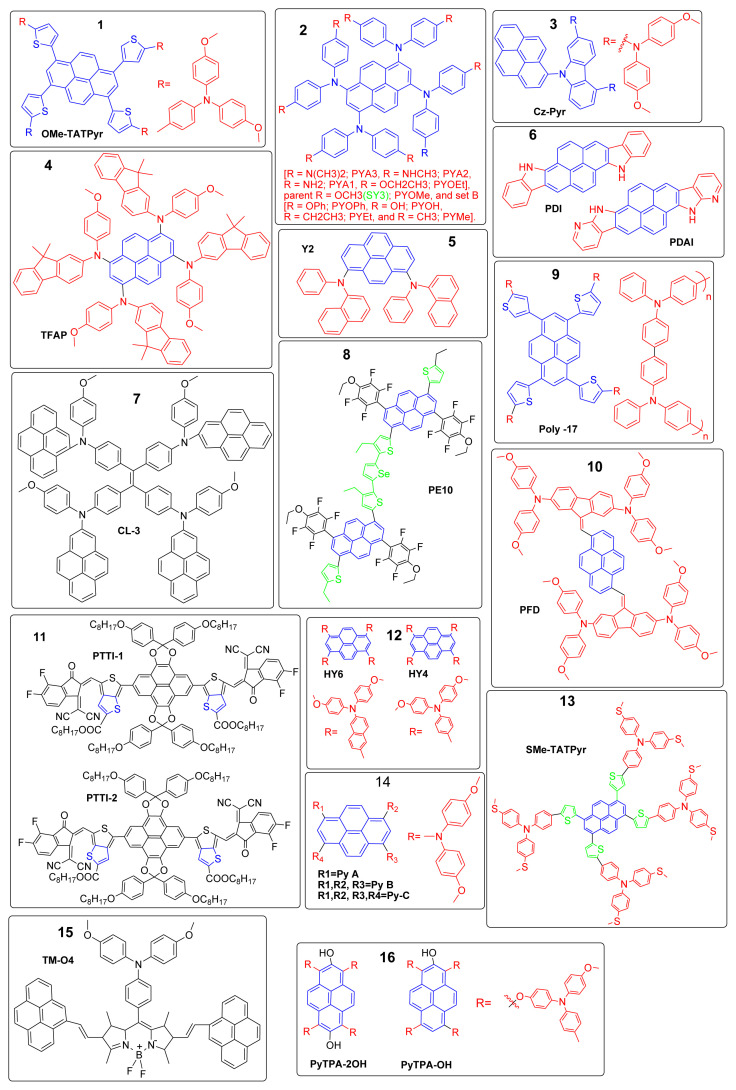
Molecular structure of the reported pyrene-based HTM. AMP: 1-ampinopyrene; OMe-TATPyr: [1,3,6,8-tetrakis[5-(N,N-di(p-methoxyphenyl)amino-p-phenyl)-thiophen-2-yl]; Cz-Pyr: carbazole-based pyrene; TFAP: 1,3,6,8-tetrakis[N-(p-methoxyphenyl)-N’-(9,9’-dimethyl-9H-fluoren-2-yl)-amino]pyrene; PDI: pyrenodiindole; PDAI: pyrenodi-(7-azaindole); PE10: fluoro-substituted pyrene into chains of polymers of thiophene and selenophene; CL-3: pyrene-based aryl amine; SMe-TATPyr: 1,3,6,8-tetrakis[5-(N,N-di(p-(methylthio)phenyl)amino-p-phenyl)-thiophen-2-yl]pyrene; HY6: (6,6′,6′’,6′”-(pyrene-1,3,6,8-tetrayl)tetrakis(N,N-bis(4-methoxyphenyl)naphthalen-2-amine); TM-04: BODIPY-pyrene-triarylamine (BODIPY-boron-dipyrromethene).

When used in PSCs, these pyrene-core-based HTMs functioned exceptionally well and had a higher PCE than control devices that use the conventional spiro-OMeTAD as the HTM. The devices with PyTPA-2OH had the best performance. Its average PCE of 23.44% (PCE_max_ = 23.50%) was the highest of all the PSCs with pyrene-core-based HTMs that have been reported thus far. This study provided a new way to combine the pyrene core, methoxy triphenylamines, and hydroxy groups to create an HTM without dopants. Figures 4 and 5 represent the timeline for the evolution of reported pyrene based PSCs and their molecular structures, respectively.

According to the above studies, it was observed that HTMs based on pyrene show a narrow band gap and excellent electrochemical and photophysical properties. Therefore, they are being looked upon more and more as a viable option. Furthermore, PSCs (Table) using various pyrene-based hole-carrying materials exhibit an outstanding performance improvement, increasing from 12.4% to 22.3%.

## 3. Conclusion

In the last few years, PSCs have drawn much attention, being investigated by researchers because they are more cost-effective than silicon-based solar cells. Having less stability and deterioration in light, moisture, and heat were the biggest challenges for researchers in terms of making PSCs commercialized. These problems may be solved by adding an interfacial layer on top of the perovskite material. Among the various small organic compounds currently used, pyrene-based molecules have outstanding efficiency. The molecular structure of pyrene is hard and planar; it also has a high degree of charge delocalization. Pyrene-based HTMs, having different functional group can be made rapidly and cheaply. The adaptability of pyrene-based compounds, which permits modifications and functional groups, increases their potential for effective charge carrier management.

To create high-performance solar cells, numerous techniques for manufacturing and novel perovskite compounds have been developed. In recent years, great number of materials, such as AMP, OMe-TATPyr, PTTI-1, PTTI-2, PYOMe, Cz-Pyr, TFAP, PDI and PDAI, Poly-17, Y2, SY3, PE10, CL-3, PFD, SMe-TATPyr, HY6, Py-C, TM-04, PyTPA-OH, PYTPA-2OH, etc., have been developed and are used as HTMs in PSCs to achieve high PCE and stability.

This review highlighted several pyrene-derived compounds that have demonstrated significant potential as effective HTMs, thereby advancing PSC technology. The review revealed that pyrene-core decorated with extended donor moieties like thiophene and terminal end-capped materials with the donor moieties increases the PSC device’s PCE from 12.4% to 23.4% and can serve as an excellent HTM. PyTPA-2OH has 23.44% PCE (PCE_max_ = 23.50%), which is the highest PCE among the reported PSCs with the pyrene-core-based HTMs up to date. All researchers attempt to discover HTMs that improve PSC efficiency and stability. This review will be helpful for future pyrene-based HTMs in designing cheap, efficient, and stable PSCs for commercialization.

## Figures and Tables

**Table:** Electrochemical data of the PSC devices with various pyrene-based HTMs.

HTM	E_HOMO_ (eV)	E_LUMO_ (eV)	E_gap_ (eV)	PCE (%)	μ_h_ (cm^2^ v^−1^ s^−1^)	Year	Reference
AMP	−5.04	−1.95	3.09	-	-	2016	[9]
OMe-TATPyr	−5.2	−3.1	2.1	20.0	2.28 × 10^−4^	2021	[26]
PYOMe	−5.11	−1.956	−3.154	12.4–12.7	3.745 × 10^−5^	2019	[28]
Cz-Pyr	-	-	-	17.2	7.4 × 10^−6^	2020	[31]
TFAP	−5.27	−3.93	1.34	19.7	4.5 × 10^−3^	2019	[33]
PDI	−5.35	−2.82	2.53	16.1	2.7 × 10^−3^	2021	[34]
PDAI	−5.54	−3.22	2.32	16.1	2.9 × 10^−3^	2021	[34]
Poly-17	−5.4	-	-	16.5	1.0 × 10^−5^	2020	[35]
Y2	−5.5	−2.8	2.7	17.9	5 × 10^−3^	2020	[36]
SY3	−5.25	−2.74	2.51	19.08	2.18 × 10^−4^	2020	[37]
PE10	−5.34	-	-	22.3	1.46 × 10^−3^	2022	[38]
CL-3	−5.39	−2.78	2.61	20.15	-	2020	[40]
PFD	−5.20	−2.81	2.39	19.92	3.65 × 10^−5^	2021	[45]
SMe-TATPyr	−5.5	−3.2	2.3	22.3	1.0 × 10^−3^	2020	[50]
HY6	−5.35	−2.76	2.59	19.34	1.62 × 10^−4^	2022	[51]
Py-C	−5.11	−2.74	2.36	12.4	-	2013	[16]
TM-04	−4.83	−3.38	1.45	18.80	16.9 × 10^−5^	2023	[53]
PyTPA-OH PyTPA-2OH	−5.00 −4.94	−2.27 −2.19	2.73 2.25	22.5 23.5	-	2024	[54]
